# Trends in Medicines Discontinuation and Implications for Access in Brazil (2014–2024)

**DOI:** 10.1002/pds.70458

**Published:** 2026-08-22

**Authors:** Raphaella Oliveira Martins de Lima, Esther Ramos Silva, Isabel C. M. Emmerick, Danielle Maria de Souza Serio dos Santos, Isabella Piassi Dias Godói, Ana Cristina Viana Campos, Luisa Arueira Chaves

**Affiliations:** ^1^ Centro Multidisciplinar UFRJ/Macaé Federal University of Rio de Janeiro Macaé Brazil; ^2^ Division of Thoracic Surgery, Department of Surgery University of Massachusetts Medical School Worcester Massachusetts USA; ^3^ Centro Multidisciplinar UFRJ/Macaé, Núcleo de Avaliação de Tecnologia em Saúde—Gestão, Economia, Educação em Saúde e Serviços Farmacêuticos (GEESFAR/NATS/UFRJ) Federal University of Rio de Janeiro Rio de Janeiro Brazil; ^4^ Laboratório e Observatório em Vigilância & Epidemiologia Social Universidade Federal do Sul e Sudeste do Pará (LOVES/Unifesspa) Pará Brazil

**Keywords:** access to medicines, Brazil, discontinuation, essential medicines

## Abstract

**Purpose:**

The World Health Organization (WHO) began recognizing drug shortages as a global issue in 2014, impacting health systems and patient care. Shortages arise from supply chain disruptions, production issues, and mismatches between supply and demand, aggravated by insufficient mechanisms for forecasting and communication.

**Methods:**

The study analyzed medicine discontinuation notifications in Brazil from 2014 to 2024 from a public secondary database. Data was downloaded, organized, and cleaned using Excel, incorporating ATC classification and verification of medicines listed on the National Essential Medicines List (NEML/RENAME). Statistical analyses included descriptive statistics and association tests (Pearson's *χ* ^2^, Fisher's exact test) examining discontinuation types, registration categories, reasons, and NEML presence.

**Results:**

Between 2014 and 2024, Brazil had 23, 110 discontinuation notifications, of which 15, 599 (67.5%) were temporary, and 7,511 (32.5%) were definitive discontinuation. The annual distribution was not homogeneous. Generic had the highest proportion of discontinuations (37.2%), surpassing branded generics (33.2%) and originator brands (15.9%). In contrast, biological products, herbal medicines, dynamized medicines, and specific medicines together accounted for only 13.73% of the total. Regarding the reasons for discontinuation, commercial reasons predominated, accounting for 73.4% of notifications. Finally, it was found that the majority of discontinued drugs were not included in NEML (73.9%).

**Conclusions:**

Medicine discontinuation in Brazil from 2014 to 2024 is mainly temporary and occurs for generics and branded‐generics due to commercial decisions. The findings highlight the complexity of supply continuity and the importance of strategies that include diversifying supply sources, strengthening regulatory oversight, and improving monitoring and communication systems.

## Key Points

- Medicine discontinuation in Brazil (2014–2024) is predominantly temporary, for generic and branded generics, and caused by commercial decisions, rising concerns for access to medicines in the country.
- Discontinuations affect essential medicines, posing risks to patient safety and continuity of care.
- Economic and market factors are major drivers of discontinuation, reflecting wider structural issues in the global pharmaceutical market.
- The study provides detailed evidence on the medicines and therapeutic groups most impacted, guiding targeted policy and regulatory responses.
- Strengthening supply monitoring, prevention strategies, and incentives for essential medicines production is crucial for improving health system resilience in Brazil.

## Plain Language Summary

Drug shortages impact access to medicines and health care. In recent years, this has been a concern for health systems and has been stated as a global health problem by the World Health Organization. So, this study analyzed official medicines discontinuation notifications in Brazil to reflect on supply chain fragilities that could impair access to medicines in the country. Between 2014 and 2024, there were many interruptions in the supply of medicines in Brazil, mostly temporary but sometimes definitive. These disruptions were most often observed for generics and medicines that people rely on to treat common health conditions. Discontinuations can indicate shortages that lead to delayed or changed treatments and even harm patient safety. By identifying the main medicines affected, the study findings highlight the need for better systems to monitor discontinuations, diversify supply sources, and encourage production of important medicines.

## 1 Introduction

In 2014, the World Health Organization (WHO) recognized drug shortages as a global problem, with relevant repercussions for health systems and patient care [1]. However, only in 2017 did the organization systematize a technical definition of the phenomenon, from two approaches: supply and demand. From the supply perspective, a shortage is characterized by an insufficient supply of essential medicines, health products, or vaccines, in a magnitude unable to meet the population's needs. These shortages may originate at different stages of production, logistics, or the distribution chain. From the demand perspective, the concept of stock‐out refers to situations in which demand exceeds availability at the point of care, resulting in localized supply disruptions and medicine scarcity [2, 3].

Medicine shortages pose a significant clinical problem, as they disrupt healthcare continuity, compromise therapeutic outcomes, and increase patient safety risks. These effects become even more concerning when scarcity affects essential medicines used as first‐line therapies for high‐prevalence illnesses or for more complex clinical conditions [4, 5].

This problem stems from the convergence of multiple determinants that interact dynamically across the pharmaceutical chain. Among the most relevant factors are economic and market conditions, the reduced profitability associated with the production and commercialization of essential medicines, as well as the increasing centralization of active pharmaceutical ingredients and finished products. This productive configuration restricts the supply capacity and increases the system's susceptibility to failures and interruptions, especially in contexts of industrial or geopolitical instability [3, 6].

Therefore, several countries have implemented policies, regulatory systems, and proactive monitoring mechanisms focused on resolving or mitigating the problem. Israel requires advance notification of possible shortages by marketing authorization holders, has established mandatory minimum stocks, and accelerated drug registration pathways to ensure alternatives in case of shortages [7]. In Canada, there is a Multi‐Stakeholder Steering Committee on Drug Shortages (MSSC) and the creation of mandatory discontinuation and shortage notification regulations [8]. The International Pharmaceutical Federation (FIP) proposes structured reporting [9]. In the United States, a regulatory model centered on mandatory notification of production interruptions, continuous monitoring, and the adoption of emergency mitigation measures is used [10]. A study points to international convergence around strategies such as early warning systems, mandatory notification mechanisms, intersectoral governance, and stock coordination instruments in high‐income countries. These initiatives remain predominantly reactive and focused on the final links of the chain, evidencing the need for preventive interventions in the initial phases of production and planning [11].

Finally, in the Brazilian context, access to essential medicines constitutes a right of the population and must be guaranteed by the State [12]; however, the management of shortage risk relies almost exclusively on a single instrument: the drug discontinuation panel, used for the analysis of notifications of interruption and reactivation of drug manufacturing [13]. According to the Resolution RDC no. 18/2014, issued by the Brazilian Health Regulatory Agency (ANVISA), manufacturers are required to notify the production/importation discontinuation, whether it be temporary or definitive [14]. This strategy, although relevant, evidences a gap in the adoption of integrated prevention and response policies that consider the entire supply chain. Furthermore, differently from other countries, Brazil adopts discontinuation monitoring and not shortages to monitor the medicines supply chain in the country. In countries like Brazil, which is heavily dependent on imports [3, 15], analyzing discontinuities can help prevent shortages and guarantee access to essential medicines. Accordingly, this study aims to analyze the national drug discontinuation panel to characterize the magnitude and patterns of medicine discontinuation, identify the main underlying reasons, and assess which medicines have been most affected, considering the potential risk of shortages in Brazil.

## 2 Methods

This is a quantitative, observational, and retrospective study using a publicly accessible secondary database, covering the years 2014–2024. The database used was ANVISA's panel on the discontinuation of medicines, which supports the management and analysis of notifications regarding discontinuation and reactivation of manufacturing and importation of medicines in Brazil [13]. The panel includes information such as subject of petition, type of discontinuation, date of petition, date of reactivation, reason for discontinuation, company name, product name, active ingredient, therapeutic class, registration number, and presentation description. The “subject of petition” field allows identification of the regulatory category of the discontinued medication, namely: generic, branded generic, specific, originator brand, biological product, dynamic, or herbal medicine. The “type of discontinuation” field indicates if the interruption is temporary or definitive. Finally, the “reason” field includes options such as increased demand, commercial motivation, issues related to the manufacturing park, logistical problems, manufacturing process, and/or availability of the active ingredient.

The database was downloaded, cleaned, and organized using Excel. Variables derived from the original dataset were created, including the year and month of petition, year of reactivation, presence in the NEML (RENAME), and ATC code. The column “code” was inserted with the international Anatomical Therapeutic Chemical (ATC) classification [16]. These data were extracted from the original “therapeutic class” column. For records where this column was blank, information was obtained from the WHO Collaborating Centre for Drug Statistics Methodology [17]. In addition, a column called “listed in RENAME” was added, in which all drugs were checked to verify if they were present on the 2024 official National Essential Medicines List (NEML, named RENAME in Brazil) [18].

An annual percentage was created to measure market discontinuations against the total number of pharmaceuticals commercialized in the country. Each registration category (generics, branded generics, originator brands, and biologics) uses the number of discontinuation notifications as the numerator, while the total number of medicine presentations commercialized in that year is the denominator, based on data from the national regulatory agency (CMED/ANVISA) [19]. Due to the unavailability of 2014 data, 2015 data were used as the denominator for that year.

### 2.1 Data Analysis

Statistical analysis was conducted in three stages. In the descriptive stage, absolute and relative frequencies (*n*, %) were estimated by type of discontinuation (temporary/definitive), year, registration category, reason for discontinuation, and presence in the NEML. Afterward, the association between the type of discontinuation and (i) registration category, (ii) reasons, and (iii) presence in RENAME was evaluated using Pearson's *χ* ^2^ test. When low expected frequencies were observed, Fisher's exact test was applied, with a significance level set at *α* = 0.05.

To characterize the therapeutic profile, medicines discontinaution reports were ranked according to the 2nd level of the ATC (Anatomical Therapeutic Chemical) code. For each regulatory category, the 10 most frequently reported codes were presented. Ranking followed a descending order; in the event of ties, the aggregate total of notifications over the period was used as the tiebreaker.

## 3 Results

Between 2014 and 2024, Brazil recorded 23, 110 discontinuation notifications, with 15, 599 (67.5%) being temporary and 7511 (32.5%) definitive. The annual distribution varied, peaking in 2017 (13.2%), 2019 (12.3%), and 2020 (11.7%), while 2014 (5.0%) and 2021 (5.7%) had lower frequencies. Generic drugs led discontinuations at 37.2%, followed by branded generics (33.2%) and originator brands (15.9%). Commercial reasons were cited in 73.4% of cases, with active ingredients (11.3%) and manufacturing processes (6.7%) being secondary. Notably, 73.9% of discontinued drugs were not on the NEML. Temporary discontinuities consistently dominated, ranging from 76% to 56%, whereas definitive discontinuations varied between 44% and 21%, with the lowest in 2021 (Table 1).

**TABLE 1:** Medicines discontinuation characteristics in Brazil, from 2014 to 2024.

Discontinuation type	*n*	%
Temporary	15 599	67.5
Definitive	7511	32.5
Year		
2014	1146	5.0
2015	2136	9.2
2016	1890	8.2
2017	3054	13.2
2018	1985	8.6
2019	2844	12.3
2020	2698	11.7
2021	1320	5.7
2022	2302	10.0
2023	1594	6.9
2024	2141	9.3
Registration category		
Generic	8606	37.2
Branded Generics	7671	33.2
New medicine	3663	15.9
Biological product	803	3.5
Specific	1819	7.9
Dynamized	5	0.0
Herbal drug	543	2.3
Reason for discontinuation		
Commercial	16 971	73.4
Industrial facility	894	3.9
Active ingredients	2615	11.3
Production process	1547	6.7
Logistical issues	623	2.7
Increased demand	90	0.4
Not reported	370	1.6
Included in NEML(RENAME)		
No	17 071	73.9
Yes	6039	26.1

**FIGURE 1:**
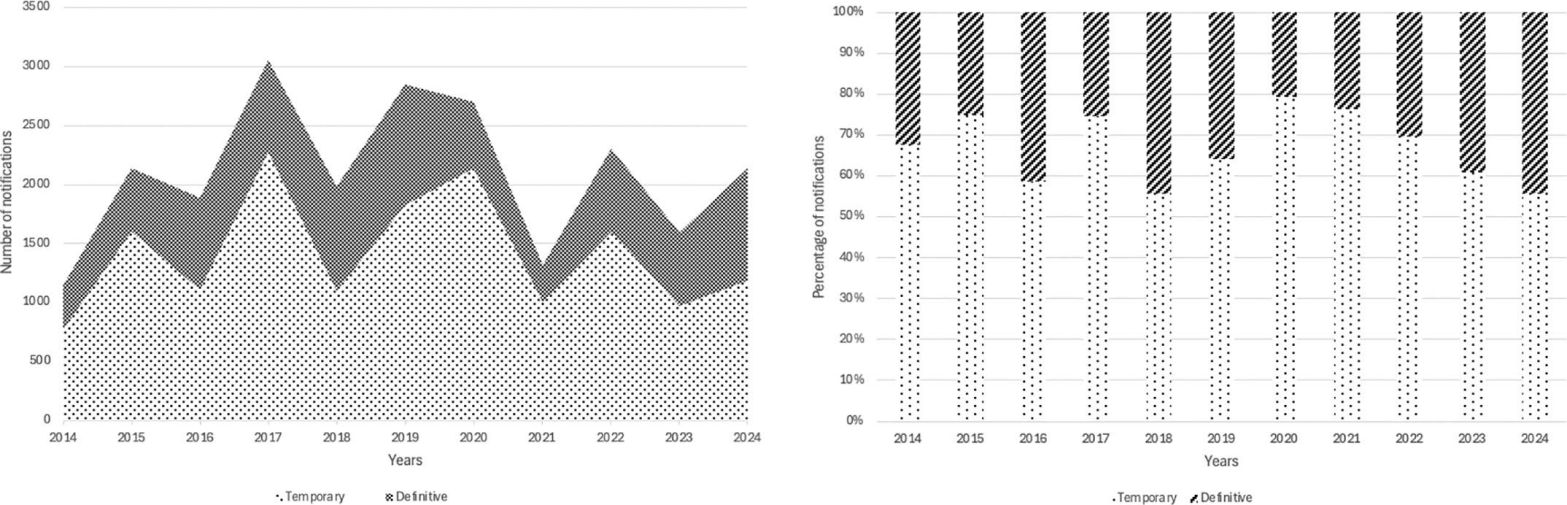
Number and percentage of medicines temporary and definitive discontinuation notification by year in Brazil, from 2014 to 2024.

**FIGURE 2:**
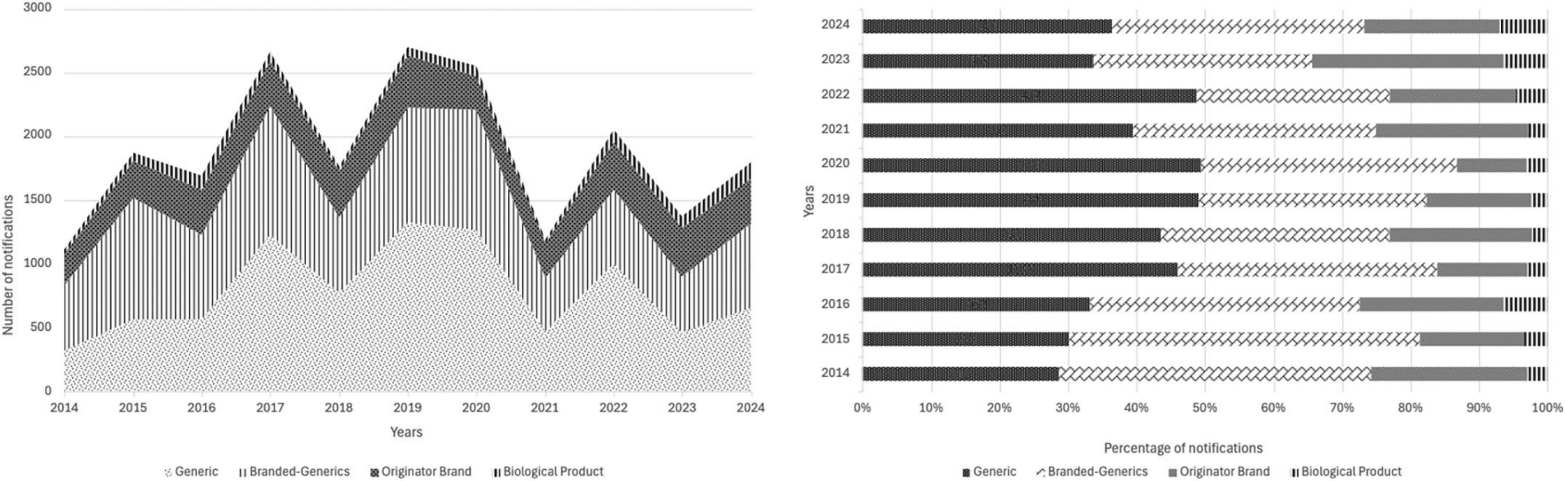
Number and percentage of medicines discontinuation notifications by registration category, by year, in Brazil from 2014 to 2024.

Figure 1 presents the number and percentage of medicine discontinuation notifications over the years, by registration category. Originator brands and biological products present low values, accounting for 28% to 10% and 2%–7% of all notifications, respectively, with a drop in 2019 (15%) and 2020 (10%) of originator brand notifications. The categories of generic and branded generic drugs, on the other hand, concentrate the largest proportion of notifications, ranging from 49% (2019 and 2020) to 29% (2014) and 51% (2015) and 28% (2022), respectively. Until approximately 2016, a predominance of branded generic drug notifications over generic drugs was observed. From this period onwards, generic drugs started to present superior values, except for 2024 (36% generics and 37% branded generics). In Figure 2, the registration categories of dynamized, specific, and herbal medicines are not presented due to a low number of discontinuation notifications (*n* = 2367, 10%) and their specificity.

Comparing the percentage of active registrations in the country with discontinuation reports per category (Table 2), it is possible to see that it follows the proportion of registries, with apparently fewer discontinuations than registries for originator brands. In Table 3, it is noted that temporary discontinuations were more numerous across all categories, but definitive discontinuations varied significantly, especially in originator brands, which had a higher proportion of definitive (24.0%) compared to temporary (14.8%). Generic and branded generic drugs showed more balanced distributions. Additionally, 78.3% of definitive discontinuations were not listed in NEML, compared to 71.7% for temporary discontinuations. Association tests indicated a statistically significant difference, suggesting that the distributions of discontinuations were not random concerning the variables studied.

**TABLE 2:** Percentage of national registrations and discontinuation reports per regulatory category from 2014 to 2024, Brazil.

Years	Generics		Branded generics		Originator brand		Biologics	
	Descontinuations	Registrations	Descontinuations	Registrations	Descontinuations	Registrations	Descontinuations	Registrations
2014^a^	20.9%	31.2%	33.4%	37.3%	16.5%	21.1%	2.2%	3.2%
2015	21.1%	31.2%	35.8%	37.3%	10.7%	21.1%	2.4%	3.2%
2016	21.0%	32.8%	24.8%	35.4%	13.3%	20.8%	4.0%	3.8%
2017	32.0%	32.8%	26.4%	34.4%	9.2%	21.3%	2.0%	4.1%
2018	26.8%	33.8%	20.6%	33.9%	12.7%	20.4%	1.4%	4.4%
2019	34.3%	33.2%	23.2%	33.7%	10.6%	20.1%	1.7%	4.4%
2020	38.7%	33.0%	29.1%	32.5%	8.0%	19.7%	2.4%	3.9%
2021	28.%	33.5%	25.8%	32.5%	16.4%	18.9%	2.0%	4.0%
2022	33.3%	33.5%	19.3%	32.3%	12.6%	19.3%	3.1%	4.4%
2023	20.8%	33.1%	19.7%	32.2%	17.2%	19.2%	4.0%	4.7%
2024	21.1%	34.3%	21.4%	32.1%	11.5%	18.6%	4.0%	4.8%

^a^ Due to the absence of available registration data for the year 2014, data from the year 2015 was utilized for analysis.

**TABLE 3:** Type of discontinuation by registration category, reason, and presence in NEML in Brazil from 2014 to 2024.

	Discontinuation type		*p* ^a^
	Temporary	Definitive	
	*n* (%)	*n* (%)	
Registration category			< 0.001
Generic	6315 (44.1)	2291 (35.6)	
Branded generic	5287 (36.9)	2384 (37.1)	
Originator brand	2119 (14.8)	1544 (24.0)	
Biological product	593 (4.1)	210 (3.3)	
Reason for discontinuation			< 0.001
Commercial	9902 (63.5)	7069 (94.1)	
Industrial facility	824 (5.3)	70 (0.9)	
Active ingredients	2352 (15.1)	263 (3.5)	
Production process	1497 (9.6)	50 (0.7)	
Logistical issues	616 (4.0)	7 (0.1)	
Increased demand	87 (0.5)	3 (0.0)	
Not reported	321 (2)	49 (0.7)	
Included in NEML (RENAME)			< 0.001^b^
No	11 188 (71.7)	5883 (78.3)	
Yes	4411 (28.3)	1628 (21.7)	

^a^ *χ* ^2^ association test.

^b^ Correction with Fisher's exact test.

Generic and branded generic medicines primarily experience discontinuations due to commercial factors, accounting for 69.1% and 71.9% of temporary interruptions, and 95.5% and 97.3% for definitive ones (Table 4). Manufacturing issues are less impactful, especially for definitive interruptions, indicating a strong influence of market factors on off‐patent medicine withdrawals. In contrast, originator brands have only 23.8% of temporary discontinuations due to commercial reasons, with production‐related issues contributing more significantly. For definitive discontinuations, commercial reasons increase to 88.4%. Biological products show a similar trend, with commercial factors leading to definitive interruptions at 94.8%, while temporary interruptions reflect a more complex split among various manufacturing causes.

**TABLE 4:** Reasons for discontinuity by type and regulatory category, 2014–2024, Brazil.

Reason for discontinuation	Commercial	Industrial facility	Active ingredients	Production process	Logistical issues	Increased demand	Various	Not reported	Total
Type of discontinuation									
Generic									
Temporary (%)	4,364 (69.11)	168 (2.66)	1087 (17.21)	528 (8.36)	67 (1.06)	14 (0.22)	11 (0.2)	76 (1.20)	6,315 (100%)
Definitive (%)	2,188 (95.50)	10 (0.44)	84 (3.67)	0 (0.00)	2 (0.09)	0 (0.00)	1 (0.0)	7 (0.31)	2,291 (100%)
Branded‐generics									
Temporary (%)	3,800 (71.88)	215 (4.07)	759 (14.36)	437 (8.27)	44 (0.83)	4 (0.08)	11 (0.1)	21 (0.40)	5287 (100%)
Definitive (%)	2,317 (97.31)	3 (0.13)	52 (2.18)	9 (0.38)	0 (0.00)	0 (0.00)	0 (0.00)	3 (0.13).	2,384 (100%)
Originator brand									
Temporary (%)	504 (23.79)	290 (13.69)	372 (17.56)	413 (19.49)	347 (16.38)	44 (2.08)	4 (0.19)	145 (6.84)	2,119 (100%)
Definitive (%)	1,365 (88.41)	9 (0.58)	117 (7.58)	36 (2.33)	4 (0.26)	3 (0.19)	0 (0.00)	10 (0.65)	1,544 (100%)
Biological product									
Temporary (%)	217 (36.59)	49 (8.26)	89 (15.01)	59 (9.95)	110 (18.55)	25 (4.22)	0 (0.00)	44 (7.42)	593 (100%)
Definitive (%)	199 (94.76)	2 (0.95)	8 (3.81)	0 (0.00)	0 (0.00)	0 (0.00)	0 (0.00)	1 (0.48)	210 (100%)

**FIGURE 3:**
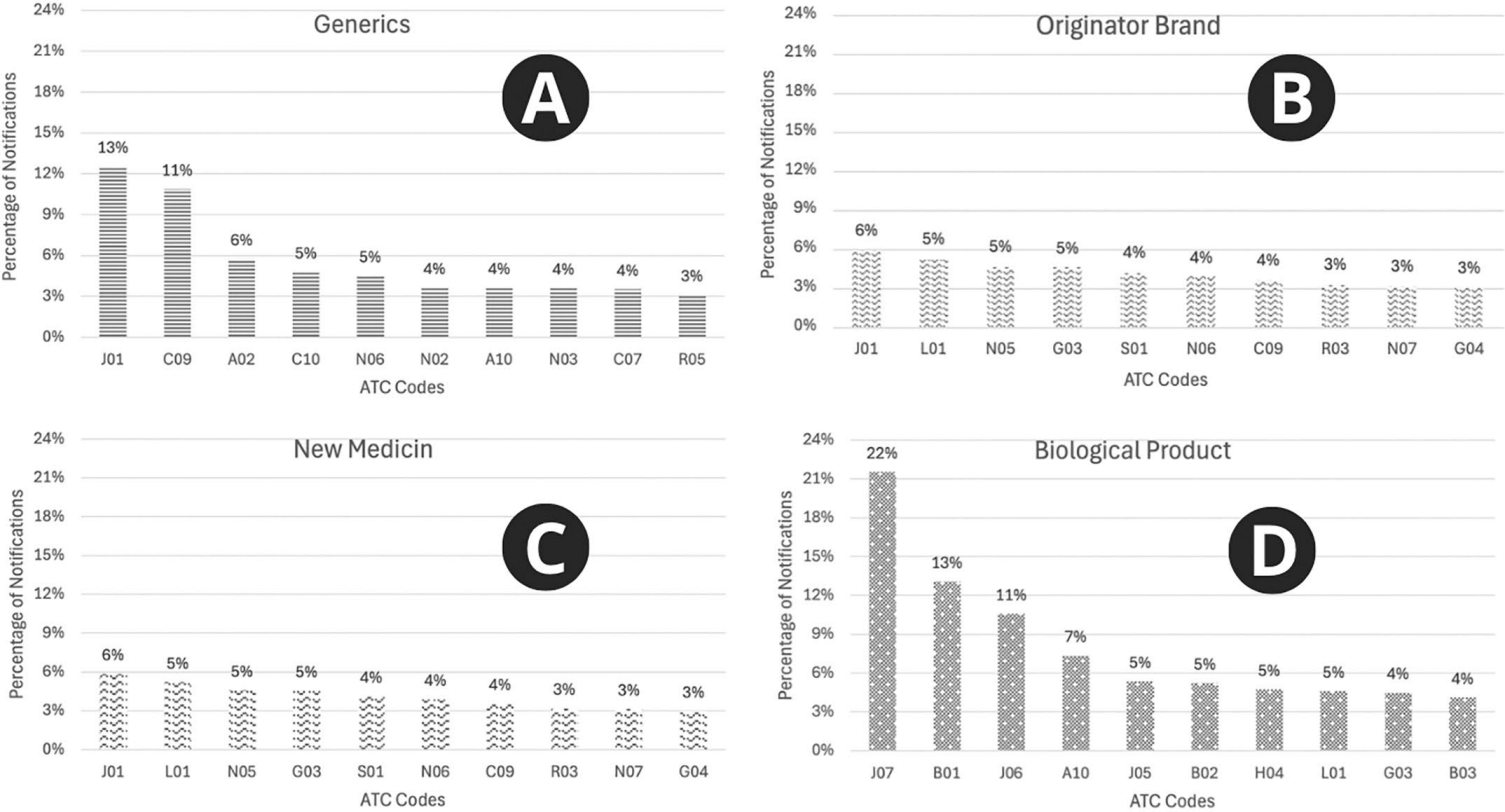
Number and percentages of top 10 ATC codes discontinued by registration category in Brazil, from 2014 to 2024. (A) Generic, (B) Branded generics, (C) Originator Brand, (D) Biological medicines. ATC codes: A02 (Medications for acid‐related disorders), A10 (Medications used in diabetes), B01 (Antithrombotics), B02 (Antihemorrhagics), B03 (Antianemic preparations), C07 (Beta‐blockers), C09 (Agents acting on the renin‐angiotensin system), C10 (Lipid‐modifying agents), G03 (Sex hormones and modulators of the genital system), G04 (Urological products), H04 (Pancreatic therapy), J01 (Antibacterials for systemic use), J05 (Direct‐acting antivirals), J06 (Immunoserums and immunoglobulins), L01 (Antineoplastic agents), M01 (Anti‐inflammatory and antirheumatic agents), N02 (Analgesics), N03 (Antiepileptics), N05 (Psycholeptics), N06 (Psychoanaleptics), N07 (Other nervous system drugs), R03 (Medicines for obstructive airway disorders), R05 (Antitussives and mucolytics), and S01 (Ophthalmologicals).

Figure 3 presents an overview of the drug discontinuation profile according to their therapeutic class by registration categories, from 2014 to 2024. Comparing the four categories, it is observed that generic (A) and branded generic (B) drugs present quite similar profiles, with emphasis on codes J01 (antibacterials for systemic use) and C09 (agents acting on the renin‐angiotensin system), which occupy, respectively, the first and second positions among the most discontinued medicines (Figure 1). In addition, these two groups share the presence of other relevant codes, such as N02 (Analgesics), N06 (Psychoanaleptics), A02 (Drugs for acid‐related disorders), C07 (Beta‐adrenergic blockers), R05 (Antitussives and mucolytics), and C10 (Lipid‐modifying agents), although distributed in different orders.

Regarding originator brands (C), a large reduction in the total number of notifications is observed when compared to the generic and branded generic categories. Even so, code J01 (antibacterials for systemic use) remains the most frequent, followed by L01 (antineoplastic agents), N05 (psycholeptics), G03 (sex hormones and modulators of the genital system), and S01 (ophthalmological preparations). The presence of antineoplastic and hormonal drugs among the most discontinued in this category indicates a distinct profile. Regarding biological drugs (D), a reduction in the total number of notifications is also observed, where codes B01 (antithrombotics) are mainly concentrated, followed by J06 (immunosuppressants and immunoglobulins) and A10 (drugs used in diabetes), in addition to other codes with lower frequency, evidencing that biological drugs present a more concentrated pattern.

## 4 Discussion

Our results show that medicine discontinuation in Brazil follows a profile. In the majority of cases, it occurs in generic or branded generic products, is temporary, due to commercial reasons, and is mainly indicated for infection treatment and important noncommunicable diseases, such as cardiovascular diseases and mental health. It is also important to note the large contribution that medicines for diabetes and cancer make to the discontinuation of originator brands and biologic medicines.

Most of the medicines discontinued in the country target the diseases that impose the greatest burden in Brazil [20], and some belong to NEML. It is worth noting J01 medicines (antibacterials for systemic use), which are extremely important for public health and have a long history of shortages throughout the world [21, 22], reflecting a problem widely recognized at the global level. The shortage of these essential medicines has been associated over the years with the concentration of production among a few manufacturers, the low economic attractiveness of the segment, and failures in the coordination between supply and demand, factors consistently highlighted in the international literature [23, 24, 25, 26, 27, 28].

In addition, the therapeutic classes reported, as well as discontinuation reasons and market categories, are consistent with the main shortage and discontinuation reports in both the scientific and grey literature, further highlighting the global magnitude of the problem [29, 30].

Overall, two distinct discontinuation profiles emerged. Generic and branded generic medicines were characterized by a strong predominance of commercial reasons, particularly among definitive discontinuations, whereas originator medicines and biological products showed a more diversified distribution for temporary interruptions, with manufacturing‐ and supply‐related factors playing a comparatively larger role.

The present findings point to market dynamics as a structural determinant of drug scarcity, reaffirming the central role of economic and productive factors in decisions to discontinue products [3, 20]. Some authors investigated predictors for shortage occurrence in the United States and Canada and found that low‐priced generics were at high risk [25, 31], in confluence with our findings. However, despite some authors declaring that generics and branded generics are more vulnerable to shortages due to low‐margin profits and, so, lack of financial incentives to stay in the market [26, 27], there is a lack of evidence to support it. Hill, Barber and Gotham [28] demonstrated that many medicines bought in tenders by South Africa, India and England had higher prices than the estimated production cost. Consequently, it is essential to conduct further research on production costs and selling prices in order to substantiate this claim. Achieving greater transparency regarding the costs associated with pharmaceutical production is fundamental to this endeavor.

Moreover, the prevalence of commercial reasons cited in discontinuity reports raises significant regulatory concerns. The market authorization holder retains the authority to determine the information disclosed in these reports, without any obligation to provide substantiating evidence. Consequently, they may categorize a discontinuation under the umbrella of “commercial reasons,” which remains a broad and nonspecific classification.

Similarly, some medicine discontinuation reports in Brazil were on the NEML, as investigations conducted in countries such as the United States, Canada, China, and Finland have also demonstrated [25, 30]. A substantial portion of scarcity involves items included in various NEML, reimbursement schemes, and the World Health Organization's Model List of Essential Medicines. In some contexts, these proportions are even higher than those observed in Brazil, indicating the vulnerability of essential medicines to discontinuation and shortage [32, 33].

The temporal analysis, in turn, revealed marked variations in the number of notifications among the studied years. Evidence from research conducted in North America and Europe, although based on distinct methodologies and periods, reinforces that this variability is not exclusive to the Brazilian context [34, 35]. The significant reduction in notifications observed in 2021 might be due to price fluctuations during the COVID‐19 pandemic. Costa and Ruas [36] found that discontinued products experienced a price increase in Brazil in 2021 compared to 2020. This may have motivated marketing authorization holders to remain in the Brazilian market, thereby reducing discontinuation notifications during that year.

Many discontinued medicines were generics vital for pharmaceutical access in Brazil, including those on the NEML. This highlights the need for sustained public policies and investments to reduce external dependence, ensure supply stability, and prevent treatment interruptions. In this sense, the National Strategy for the Development of the Health Economic‐Industrial Complex (CEIS) reflects the current Brazilian government's commitment to reindustrialization through the integration of health, industrial, science, and technology policies. The health sector accounts for approximately 10% of Brazil's Gross Domestic Product. Still, Brazil's health trade deficit has increased substantially, by approximately 80% over the past decade, largely driven by dependence on imported high‐value‐added products. To address this, the strategy foresees R$ 42 billion in investments by 2026, with R$ 23 billion expected from the private sector, aimed at strengthening domestic production capacity [37, 38].

This analysis is one of the first of the Brazilian discontinuation report system in the scientific literature. Costa and Ruas [36] have also analyzed it; however, for a shorter period of time (2018–2022) and to investigate the relation between discontinuation reports, prices, and the influence of the COVID‐19 pandemic. Both analyses showed the predominance of generic, cardiovascular, antibiotic medicines and temporary discontinuations. While our analysis demonstrated the notification patterns across different registration categories over a longer period of time. It reveals patterns and vulnerabilities in the pharmaceutical supply chain, serving as a strategic tool for better public policies to ensure access to essential medicines. However, 42.29% of temporary reports lacked a return date, indicating either prolonged discontinuations or regulatory flaws in reporting requirements.

All these problems show that Brazil has much more to do in the regulation of shortages. First, it needs to have a real shortage notification system to help health managers and caregivers understand medicine availability and shape their practices effectively, so they can continue patient care treatments with minimal harm when facing a shortage. Second, as a country that ensures access to medicines as a citizenship right [39], it is fundamental that it has an instrument that allows stakeholders to understand the national pharmaceutical supply chain, especially regarding medicines on the NEML. Without that knowledge, it is impossible to ensure access to medicines and to propose public policy to enable it.

The main limitation is that our data refer to discontinuation reports and not shortages, as in Brazil, we do not have a national shortage notification database. Discontinuation is when the product's national marketing authorization holders decide to stop production or importation to Brazil. This does not necessarily mean it will have a shortage since it depends on the number of other registrations and their relevance to the Brazilian market share. However, this can be used to discuss the pharmaceutical supply chain and demonstrate which type of medicines are leaving the Brazilian market and thus may cause shortages in the country. Therefore, we compared our data with medicines shortages scientific literature.

The results show that medicine discontinuation in Brazil is a recurring issue that affects access to essential treatments, primarily due to commercial and production reasons. While most interruptions are temporary, their frequency highlights ongoing supply instability, risking shortages. This study emphasizes the need to view medicine discontinuation as a public health issue, as it threatens the constitutional right to access medicines in Brazil.

## Funding

Raphaella Oliveira Martins de Lima received an undergraduate scientific scholarship to work on this project from the Council for Scientific and Technological Development (CNPq).

## Ethics Statement

As this is a study based on publicly available secondary databases, it was exempted from ethical review and informed consent requirements.

## Conflicts of Interest

The authors declare no conflicts of interest.

## Data Availability

The data that support the findings of this study are available in Painel de Descontinuação de Medicamentos da ANVISA at https://www.gov.br/anvisa/pt‐br/acessoainformacao/dadosabertos/informacoes‐analiticas/descontinuacao‐de‐medicamentos. These data were derived from the following resources available in the public domain: ANVISA Website, https://sad.anvisa.gov.br/MicroStrategy/servlet/mstrWeb.
